# Children’s developing views of social excluders: A dissociation between social evaluation and partner preference

**DOI:** 10.1111/bjdp.12413

**Published:** 2022-04-19

**Authors:** Amanda Mae Woodward, Lindsay A. Horen, Sarah J. Knoll, Jonathan S. Beier

**Affiliations:** ^1^ Department of Psychology University of Minnesota, Twin Cities Minneapolis Minnesota USA; ^2^ University of Maryland College Park Maryland USA; ^3^ Massachusetts General Hospital Boston Massachusetts USA; ^4^ Independent Scholar Washington D.C. USA

**Keywords:** play preferences, social evaluation, social exclusion

## Abstract

When facing social exclusion, children seek to strengthen existing social connections and form new ones. This study asked whether they also make strategic choices about the targets of their affiliative goals. Three‐ to six‐year‐olds (*N* = 69; 36 female; mostly non‐Hispanic White) observed characters acting inclusively or exclusively. All ages viewed excluders more negatively than includers, but only five‐ and six‐year‐olds preferred includers as play partners. Despite easily detecting and remembering exclusion events, younger children expressed no play partner preference. Children's verbal justifications revealed that older children choose partners more carefully and draw on a richer understanding of exclusion. More generally, the initial dissociation between social evaluation and preference formation underscores that these are distinct processes with different developmental trajectories.

**Statement of contribution**

***What is already known on this subject?***

- Children who observe exclusion adjust their behavior to become more attractive social partners.
- Prior work has investigated children’s preferences and evaluations toward excluders and includers.
- This prior work found surprising developmental trends, but methodology constrained conclusions.

***What does this study add?***

- By 5 years, children who observe exclusion strategically select partners likely to include them.
- Younger children evaluate excluders negatively, but this does not guide their partner selections.
- With age, children justify preferences with abstract, meaningful reasoning about exclusion.

## BACKGROUND

Social exclusion puts young children at risk for a host of negative outcomes. Chronically excluded preschoolers are more likely to develop poor self‐regulation and internalizing problems, and they may become maladjusted, acting more aggressively and less cooperatively (Crick et al., 1997; Stenseng et al., 2014a, 2014b). Following these outcomes of exclusion, children may withdraw further, thereby increasing the likelihood of additional exclusion and even poorer outcomes (Rubin et al., 2009). Given the potential for this harmful cascade, it is critical to understand how children respond to social exclusion and what strategies they use to mitigate its negative effects.

Initially, young children who encounter social exclusion are motivated to become more attractive social partners. After briefly witnessing or experiencing exclusion, preschool‐aged children think more about affiliation, remember social events more accurately and attend more to the mental states of others (Marinović & Träuble, 2018; Song et al., 2015; White et al., 2016). Children concerned about exclusion also sit in closer proximity to others and imitate their actions more accurately (de Klerk et al., 2020; Marinović et al., 2017; Over & Carpenter, 2009; Watson‐Jones et al., 2014, 2016). Collectively, these affiliative responses are likely to strengthen children's existing social connections and facilitate the formation of new ones.

Yet, enhancing one's social value does not guarantee future inclusion. It is also important to select one's desired partners wisely (Kuhlmeier et al., 2014). Exclusion concerns should encourage children to direct their social efforts towards people who are likely to include them, and away from those who have a history of excluding them or similar others. Making adaptive partner choices thus requires children who are concerned about exclusion to monitor how inclusively or exclusively people have acted in the past, form social evaluations on the basis of these histories and then act in accordance with these evaluations.

Only one prior study has directly examined whether preschool‐aged children form evaluations or generate preferences towards others based on their prior acts of social exclusion and inclusion. Hwang and Markson (2020) observed that 3‐ to 6‐year‐olds were more likely to label their prior excluders than their prior includers as ‘mean’. However, when choosing social partners for a new game, only 5‐ and 6‐year‐olds took a character's history of exclusive versus inclusive play into account. Three‐ and four‐year‐old children did not avoid their prior excluders. This divergence of younger children's evaluations of players and their play partner preferences was unexpected, and suggests that exclusion may have impacted their meanness attributions, but not their partner preferences.

Dissociations between children's social evaluations and preferences are not typically found at this young age. For instance, preschoolers label prosocial characters as nicer than antisocial ones and say that they like the same prosocial characters more (Van Kenward & Dahl, 2011; de Vondervoort & Hamlin, 2017). In other research, evaluation and preference measures are combined into a single score, suggesting that the measures are tightly linked. Buon et al. (2014) created an index combining children's evaluations of which character is ‘good’ and ‘nice’ with their social preferences, such as which character they ‘like’ or ‘want to play with’. However, one recent study has also observed an unexpected divergence in children's evaluations and preferences for prosocial and antisocial characters: When focusing just on characters’ intentions, both 3‐ and 4‐year‐olds are more consistent when making choices about who is ‘nicer’ than when indicating which character they like more (Van de Vondervoort & Hamlin, 2018).

It is critical for more research to investigate children's person preferences in the context of social exclusion. Hwang and Markson (2020) noted that their study design likely induced a strong bias to play with novel characters, which may have swamped other effects in younger children; consequently, it is unclear whether children truly do not incorporate characters’ prior play histories in their partner choices until age 5. If the surprising dissociation those authors observed arose from study‐specific methodological choices, these choices should be better understood, and the full trajectory of children's preferences should be described. Alternatively, if this dissociation between evaluations and preferences can be observed separately from Hwang and Markson (2020), it may reflect cognitive or motivational factors with important implications for understanding the systems that underlie children's developing partner preferences – both after social exclusion and more broadly.

The current study investigates 3‐ to 6‐year‐old children's reactions to people who have acted exclusively or inclusively, by collecting both niceness ratings and play partner preferences from children who have witnessed exclusive and inclusive play events. Children's play partner preferences were examined by asking children to choose between a previously inclusive and an exclusive player, reducing the impact of a potential novelty bias. Because prior work suggests that younger preschoolers’ performance on evaluation and preference measures can be constrained by attentional and memory limits (Van Kenward & Dahl, 2011; de Vondervoort & Hamlin, 2017), we also assessed children's ability to detect exclusion events and track each character's history of acting exclusively or inclusively. Additionally, to further probe children's explicit reasoning about their affiliation preferences, we collected their justifications for these decisions.

We preregistered analyses of children's developing abilities to detect exclusion and to track, evaluate and form affiliative preferences towards excluders and includers (https://osf.io/wpu2v/). Because this study was initiated prior to the publication of Hwang and Markson (2020), the pre‐registration did not anticipate a dissociation between evaluations and preferences. Consequently, we also conducted a set of non‐pre‐registered, exploratory analyses to better understand the developing relation between children's exclusion‐based evaluations and partner preferences.

## METHODS

### Participants

Per our pre‐registration, we aimed to test 64 3‐ to 6‐year‐old children, but continued testing until all scheduled children had visited our laboratory. This sample size was determined using an *a priori* power analysis for a one‐tailed Wilcoxon sign rank test. This power analysis assumed an effect size of *d* = 0.32, and an 80% chance of detecting a true effect. The final sample included 69 children, divided into younger (19 female, 16 male; mean age = 48.39 months, range = 36.30–59.80) and older (17 female, 17 male; mean age = 71.27 months, range = 60.66–81.90) groups.

Children were recruited to the lab from the nearby community, tested at an on‐campus preschool, or tested onsite at a local children's museum in a small mid‐Atlantic city. All children were typically developing, had normal or corrected‐to‐normal vision and hearing, and heard English at least 50% of the time. Due to the rapid nature of on‐site testing, we did not collect additional demographic information. Recent research by our group has determined that these testing locations draw samples composed of children who are primarily non‐Hispanic White (70%) with a modal family income above $100,000. Parents of all participating children provided informed consent.

An additional 28 children participated, but were not included in the final sample. Three did not complete the study, three experienced technology errors, and two did not meet eligibility requirements. Twenty children were excluded due to an experimenter error resulting from a misunderstanding of the scripted procedure; this decision was made prior to coding or analysing their responses.

### Materials

Children viewed video stimuli on two 10.1 Lenovo tablet computers. A trio of hand‐puppets appeared in an inclusion game, and another trio in an exclusion game. Each trio wore different matching colours and included a dog (the focal character) and two mice (the includers or excluders). All characters had pronouns that matched the child's own.

In both games, two mice introduced themselves (one holding a ball) and stated that they would play a game of catch; the dog replied affirmatively. In the inclusion video, the three characters received the ball 12 times each. In the exclusion video, the two mice only threw the ball to each other; consequently, they each received the ball 18 times, and the dog never received it. Thus, both games lasted for 36 throws, long enough to induce feelings of exclusion (Hartgerink et al., 2015; Watson‐Jones et al., 2016; White et al., 2016).

### Procedure

**FIGURE 1:**
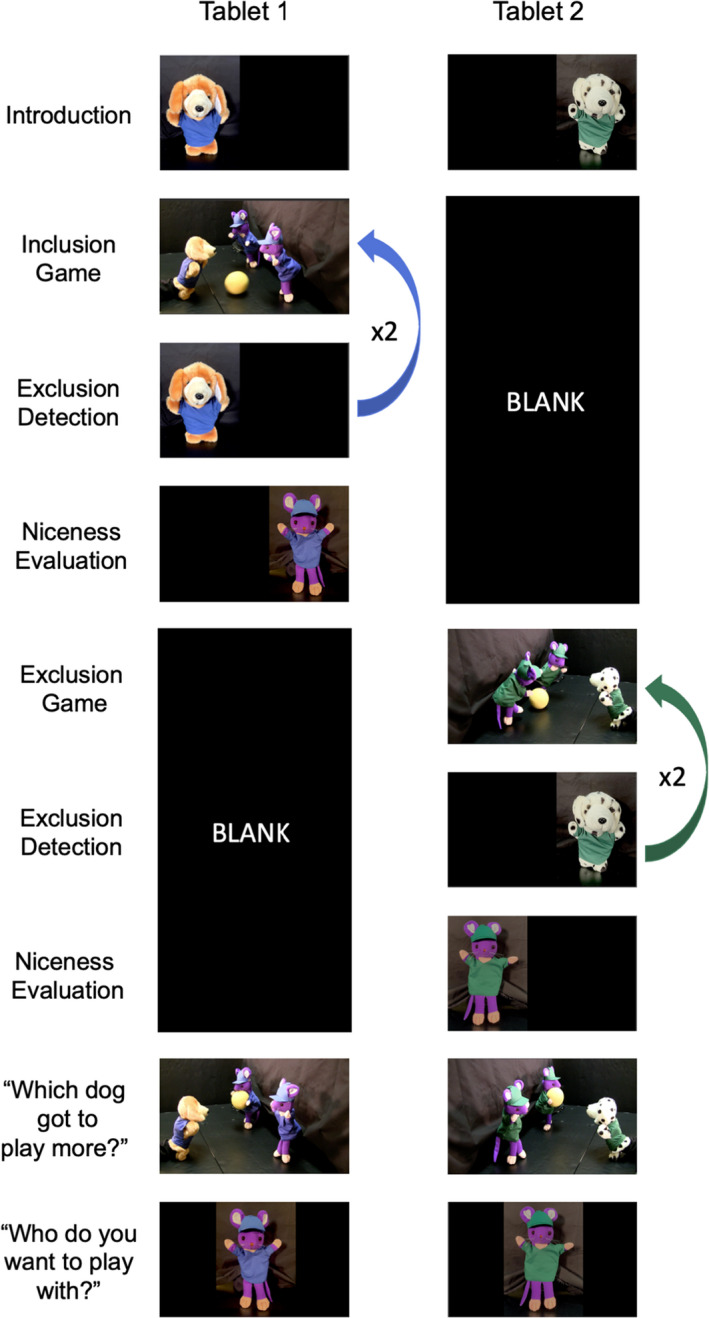
Schematic of procedure. *Note*. This schematic depicts the content displayed on the two tablets children interacted with. The order of games was counterbalanced across children

See Figure 1 for the tablet sequence. Sitting opposite the experimenter, children watched the first game, followed by the exclusion detection and social evaluation questions for that event. Then, they watched the second game and answered the same questions. Finally, the experimenter asked questions requiring children to reflect on both events together: a memory check, a play preference and probe of their justifications for their play preferences.

#### Exclusion detection

After children viewed the first game, the experimenter asked, ‘Did the dog get to play or did [he or she] have to watch’? To ensure that any child who initially failed to detect exclusion would eventually do so, and that all children would have the same amount of viewing experience before answering the remaining questions, the experimenter provided feedback on all detection responses and then replayed the same video.

#### Social evaluations

The experimenter displayed an image of one mouse from the preceding game, and asked: ‘Is the mouse nice or not nice’? Based on children's replies, the experimenter next provided a 3‐point smiley or frowny face scale for children to quantify their evaluation (e.g. ‘a little nice’, ‘nice’, ‘really nice’). Children's replies on the 3‐point scales were combined to form a 6‐point bipolar niceness scale ranging from 1 = ‘very not nice’ to 6 = ‘very nice’.

#### Memory check

The experimenter displayed one trio on each tablet. Each image featured the dog and mice just before the game of catch had begun. The experimenter asked, ‘Which dog got to play more’?

#### Partner preference

The experimenter displayed one mouse from each game and asked, ‘Which mouse would you want to play with’? Following children's selections, the experimenter probed their justifications in two ways. First, the experimenter asked, ‘Why do you want to play with [the preferred partner]’? Second, the experimenter asked a follow‐up question. Using a new display board to reference each mouse, the experimenter asked children whether their partner preference was driven by a motivation to approach their preferred partner (i.e. ‘because that mouse would play with you’) or to avoid their dispreferred partner (i.e. ‘because the other mouse would not play with you’). The experimenter also asked children to explain this response, but these comments were not analysed.

### Design

The relative order of the exclusion and inclusion games, the colour of the outfits worn by excluders and the side on which the exclusion game was presented were all counterbalanced. The including and excluding puppets were always the mice, and the dog was always the puppet who was included or excluded. All questions were asked in a fixed order.

### Coding

Two coders, blind to which dog was excluded, recorded children's responses from study session videos. Reliability was close to perfect (Cronbach's kappa = .99). Discrepancies were resolved through discussion with the blinded first author.

### Statistical approach

Per our pre‐registration, we conducted analyses on the overall sample and on the younger (3‐ and 4‐year‐old) and older (5‐ and 6‐year‐old) age groups separately. Although our pre‐registration indicated that we would only include participants who accurately answered the memory check and first exclusion detection question in the final analyses, we chose to include all participants, as very few answered these questions inaccurately (inaccurate memory check *N* = 2; failed exclusion detection *N* = 4). When these participants are removed, the results are consistent with those reported below.

We used non‐parametric analyses to understand children's responses to each question type. As all questions had clearly hypothesized answers, we used pre‐registered one‐tailed tests. To determine whether children were more likely to report having detected exclusion after it occurred, we used a McNemar test to compare children's detection responses after watching exclusion and inclusion games. To determine whether children's niceness ratings for excluders were lower than their ratings for includers, we conducted a paired Wilcoxon signed‐rank test. We used binomial tests to examine whether children's memory check responses, play preferences and reasoning for play partner preferences differed from chance (*mu* = 0.50). We expected children to remember who excluded the dog, prefer the includer and use an approach (vs. avoidance) motivation more often than chance.

Additionally, we used linear mixed models (LMMs) to assess how age related to children's responses to each question type. For the exclusion detection and social evaluation questions, we fit a model for each measure, featuring our predictors of interest (i.e. age in months, game type and their interaction), and using random intercepts for each child to compare across game types. For the memory check and play preference questions, which were not repeated measures, we fit logistic regression models featuring only age as a predictor. For both model types, we also fit preliminary models that included gender and all counterbalancing factors. If a counterbalancing factor significantly predicted the outcome, we included the significant predictor in the logistic regression with age.

## RESULTS

### Exclusion detection

Children accurately reported that social exclusion occurred after observing the exclusion event but not after observing the inclusion event, 61 of 69, χ^2^(1) = 59.02, *p* < .01. This pattern was present in both younger, χ^2^(1) = 27.03, *p* < .01, and older, χ^2^(1) = 30.03, *p* < .01, age groups. Convergent results were obtained in the GLMM: Only game type predicted children's reports that exclusion had occurred, *b* = −27.41, *z* = −4.57, *p* < .01.

### Social evaluations

Children viewed includers (*M* = 5.29, *SD* =1.32) as nicer than excluders (*M* = 2.83, *SD* = 1.98), *V* = 65.5, *p* < .01. This pattern was present in both younger (*V* = 25.5, *p* < .01) and older (*V* = 4.5, *p* < .01) children. In the GLMM, however, both Age, *b* = −0.05, *t*(131) = −3.43, *p* < .001, and the Age × Game Type interaction, *b* = 0.06, *t*(67) = 3.11, *p* = .003, were significant. Simple slope analyses revealed that evaluations of includers remained strongly positive across all ages, *b* = 0.01, *t*(67) = 0.75, *p* = .46. With increasing age, children rated social excluders as less nice, *b* = −0.05, *t*(67) = −2.97, *p* = .004.

### Memory check

Nearly all children answered the memory check correctly (67 of 69, *p* < .01). This pattern was present in both younger (33 of 35, *p* < .01) and older children (34 of 34, *p* < .01). In the logistic regression, age was not associated with children's memory check responses, *b* = 0.20, *z* = 1.43, *p* = .15.

### Play preference

Overall, children preferred the includer as a play partner (49 of 69, *p* < .01). However, this effect was driven by older children (30 of 34, *p* < .01). Younger children showed no play partner preference (19 of 35, *p* = .37). The logistic regression supported this pattern: Age significantly predicted children's partner preferences, *b* = 0.07, *z* = 2.09, *p* = .03. The preliminary logistic regression indicated that outfit colour predicted children's preferences, such that children were more likely to choose the blue mouse as a play partner, *b* = 1.26, *z* = 1.97, *p* = .04. However, when shirt colour, age, and their interaction were entered into the main model, shirt colour (*b* = −1.99, *z* = −0.59, *p* = .55) and its interaction with age (*b* = 0.06, *z* = 0.95, *p* = .34) did not predict children's play preferences.

We also examined children's reasons for choosing a specific play partner. One child gave both possible responses and was excluded from this analysis. The remaining children indicated that they chose their play partner because they thought that the chosen mouse would play with them (56 of 68, *p* < .01), rather than to avoid the mouse they did not choose. This pattern was found in both younger (28 of 34, *p* < .01) and older children (28 of 34, *p* < .01).

### Non‐pre‐registered analyses

Like Hwang and Markson (2020), we observed a dissociation between younger children's evaluations of social includers and excluders and their partner preferences. We thus conducted a series of exploratory analyses to better understand this pattern.

**FIGURE 2:**
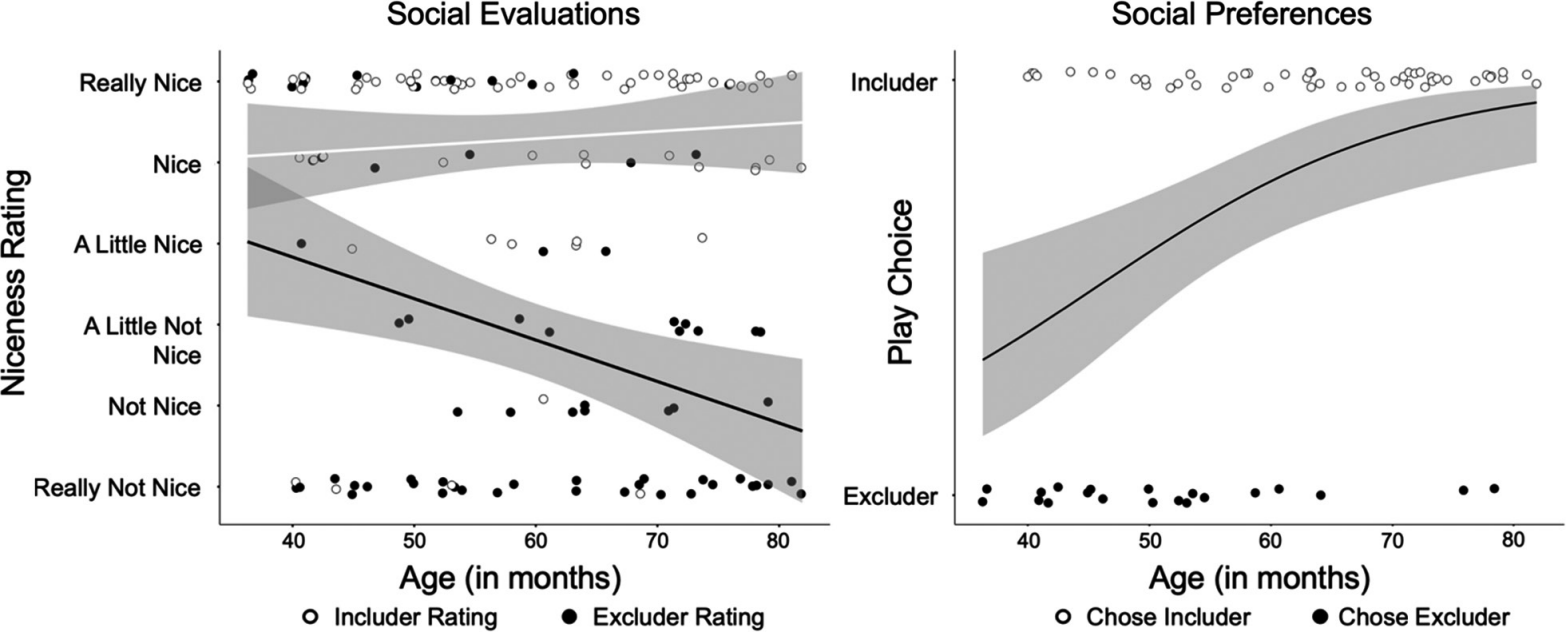
Children's social evaluations and play partner preferences. *Note*. The shaded areas depict 95% confidence intervals

Figure 2 indicates that children's evaluations of includers and excluders begin to diverge around 3.5 years, whereas they only prefer includers as play partners by about 5 years. This is supported by analyses examining these judgements separately at each year of age. For these analyses, we accounted for multiple comparisons by applying a Bonferroni correction. All reported p values have been adjusted to account for the four additional analyses. Two‐tailed Wilcoxon signed‐rank tests found no evidence that 3‐year‐olds evaluated includers and excluders differently (*p*_adjusted_ = .12); however, older children did (all *p*_adjusted's_ < .04). Two‐tailed binomial tests found no evidence that 3‐ and 4‐year‐olds preferred either character (*p*_adjusted's_ < .05); however, 5‐ and 6‐year‐olds preferred the includer (both *p*_adjusted's_ < .04).

We next considered whether there was an association between how differently children evaluated includers versus excluders and their subsequent play preferences. We constructed a difference score for children's evaluations by subtracting their niceness ratings for excluders from their ratings for includers, and then fit a logistic regression predicting play partner preferences from this difference score, age in months and their interaction. Age, χ^2^(1) = 8.35, *p* = .003 predicted play partner preferences, but the difference score and interaction term did not (both *p*'s > .18).

Finally, we examined the justifications that children provided for their play preferences. First, while being blind to children's age and stated preferences, we inductively generated content categories capturing clusters of response types from transcriptions of their comments. Second, two blind coders recorded non‐exclusive occurrences of each category (κ = .90); Table 1 reports these counts. Third, we used Fisher's exact tests to examine associations between children's age group, partner preferences and justification classifications.

**TABLE 1:** Frequency of categories in children's play choice justifications

Variable		*n*	Characteristics		Feelings		Game play		Decision making	Abstract thinking		
			Positive	Negative	Positive	Negative	Inclusion	Exclusion	Comparison	Future	Moral	Power
Age	Younger	35	10	0	8	1	4	2	2	1	0	0
	Older	34	17	1	0	0	9	4	11	3	6	6
Play choice	Includer	49	22	1	4	1	12	4	12	3	6	6
	Excluder	20	5	0	4	0	1	2	1	1	0	0

Note

Individual justifications could be classified into more than one category. Frequencies indicate the number of children mentioning each category in their response.

The categories were: social evaluation of the mice (i.e. referencing their valenced personal characteristics), preference towards the mice (i.e. referencing children's own feelings towards them), explicit mention of inclusive or exclusive acts or events, evidence for more complex decision‐making (specifically, whether children compared the two characters, as opposed to describing just one) or the invocation of more abstract concepts. This final category comprised several additional sub‐categories: future‐oriented strategizing about how a mouse would treat the child (e.g. ‘…he won't pass it to me’), using morally laden terminology (e.g. ‘…the green one doesn't share’), and describing a mouse's power to grant or deny access to gameplay (e.g. ‘he let the green dog play’).

Three justification categories were associated with age group. Younger children were more likely to reference their own feelings towards the mice (*p* < .01); however, all of these responses were simple statements that offered little insight into their partner preferences (e.g. ‘Because I like him’). Older children were more likely to compare the two characters and to provide responses revealing more abstract reasoning (both *p*'s < .01). However, none of these justification classifications was associated with children's play partner preferences (*p*'s > .09). The remaining categories, referencing game play and offering social evaluations, were not associated with age group or with play partner preferences (*p*'s > .09).

## DISCUSSION

To ensure healthy development, children must avoid social exclusion and the harm it can bring. This study found that, around 5 years of age, children incorporate information about a potential partner's history of inclusive or exclusive behaviour into their social preferences. These preferences appear to reflect children's expectations that people who have previously included others are likely to include them. This work complements earlier research showing that children who are worried about exclusion adjust their behaviour to become more attractive social partners (Watson‐Jones et al., 2016). Beyond modifying their self‐presentation, the present study suggests that 5‐year‐olds also strategically seek less risky social partners after observing exclusive and inclusive others.

This work also addresses a critical open question about whether even younger children consider information about past play behaviour when making plans for future affiliation. Like older children, children as young as 3.5 years detected exclusive behaviour and evaluated excluders negatively. However, they did not consistently prefer to play with includers over excluders. This discrepancy between younger children's social evaluations and partner preferences replicates an unpredicted observation reported by Hwang and Markson (2020). The convergent findings from our pre‐registered study, conducted independently using a different procedure, provide strong support for the developmental sequence those authors observed: From about 3.5 to 5 years of age, children recognize that prior excluders are less nice than includers, but this information does not yet impact their play partner preferences.

What might account for the dissociation between younger children's social evaluations and partner preferences? After observing a similar dissociation, Hwang and Markson (2020) noted that it might have arisen from response biases inherent to their study procedure. Participants in their study chose between a prior character and a novel character, so their apparent insensitivity to a prior character's play history may have resulted from a strong preference to play with new people. The present work asked children to choose between equally familiar includers and excluders, thereby avoiding novelty preferences. Moreover, it is unlikely that younger children's preferences were constrained by cognitive limitations such as being unable to recognize or remember what happened, as children at all ages had near‐perfect scores on the exclusion detection and memory check measures.

Dissociations between measures may arise when one task is more cognitively demanding than another, or when they rely on different cognitive processes. The evaluation measure required children to evaluate characters separately, but the preference measure involved comparing both characters. It is possible that this format was more demanding for younger children. However, the preference measure used an identical format to the memory check, which younger children answered easily. Thus, if younger children found it more difficult to express a partner preference than to share an evaluation, it is unlikely due to the question format.

We suggest that the separate trajectories of children's evaluations and preferences may instead reflect differences in how these decisions are made and the social information children at different ages find most relevant. Younger children's social preferences can be idiosyncratic, and they may fixate on features unrelated to play experiences, such as the colour of a person's outfit (as seen here; also reported by Van de Vondervoort & Hamlin, 2017). Older children, on the other hand, both preferred the includer and were more likely to justify their preferences by making a direct comparison between the prior excluder and prior includer. Although such comparisons were unassociated with play partner preference, it is notable that they were virtually absent in younger children. This suggests that older children may engage in more careful decision‐making processes when selecting play partners.

Why might older children have approached a decision about potential playmates more carefully than younger children? In the United States, where this research was conducted, children generally begin formal schooling in kindergarten at age 5 (National Center for Education Statistics, 2018). School programmes designed to promote positive relationships and to reduce bullying are common in early formal schooling years and may play a role in children's reported play preferences. Future research should examine how these programmes influence children's reported play preferences and if reported preferences differ from actual decisions about play partners.

Furthermore, kindergartners exhibit different play styles than younger children, directly engaging in more group play and spending less time as onlookers or play observers (Rubin et al., 1978). Based on these experiences, older children may encounter exclusion and its negative consequences more often and in more significant ways than younger children. Therefore, older children may experience greater concern about whether they will be included.

Advances in cognitive ability and social understanding may further support older children's consideration of a partner's play history in additional ways. For instance, older children may use more intricate social processes when forming peer preferences and weigh both positive and negative factors. Some children may be viewed as more popular because they are well liked by their peers and act in prosocial ways. Other children may be perceived as popular, but not well liked. This can occur when children are sociable, but exhibit relationally aggressive behaviour, such as bullying others (Nelson et al., 2005). Children's justifications in the current study seem to align with these more complex social processes. When justifying their preferences, older children considered how characters would treat them in the future, referenced moral norms about sharing, and described exclusion as an assertion of power. Such patterns not only indicate that older children may have more experience with exclusion but also indicate that they may think about it in more meaningful ways. Further research is needed to elucidate whether this more advanced conceptual reasoning about social exclusion relates to children's play choices. Thus, an additional contribution of the present work is its identification of three domains – the future, morality and social power – that some children spontaneously invoke when reflecting on their social preferences involving excluders. This provides a foundation for future research to investigate these considerations in a more focused manner.

More generally, this study provides an important reminder for broader research in social cognitive development: Social preferences do not always align with social evaluations. Children may recognize an individual as less nice, yet nevertheless wish to be their friend (Hawley, 2003; Nelson et al., 2005). In the present work, it is possible that older children's heightened exclusion concerns led them to weigh a character's play history more heavily, thereby bringing their evaluations and preference into apparent alignment. However, younger children's preferences reveal that social evaluation and preference formation are different processes that perform different functions. A critical goal for future work will be to provide a more comprehensive account of how the relation between children's evaluations and preferences is affected by their increasing cognitive abilities, expanding social knowledge and shifting social motivations.

## CONFLICTS OF INTEREST

The authors declare that they have no conflicts of interest.

## AUTHOR CONTRIBUTIONS

**Amanda Mae Woodward:** Conceptualization; Data curation; Formal analysis; Investigation; Methodology; Resources; Supervision; Visualization; Writing – original draft; Writing – review & editing. **Lindsay A. Horen:** Conceptualization; Investigation; Methodology; Project administration; Visualization; Writing – review & editing. **Sarah J. Knoll:** Conceptualization; Investigation; Methodology; Project administration; Writing – review & editing. **Jonathan S. Beier:** Conceptualization; Investigation; Methodology; Supervision; Writing – review & editing.

### OPEN RESEARCH BADGES

This article has been awarded Open Data, Preregistered Research Designs Badges. All materials and data are publicly accessible via the Open Science Framework at [https://osf.io/wpu2v/?view_only=4faa9f36f8fa445a8d0caa6192b6e068].

## Data Availability

The original materials and data accompanying this study are available at the Open Science Framework: https://osf.io/wpu2v/?view_only=4faa9f36f8fa445a8d0caa6192b6e068. Permission is required to use the stimuli of this study.
